# Attitudes, skills and use of evidence-based practice among UK osteopaths: a national cross-sectional survey

**DOI:** 10.1186/s12891-018-2354-6

**Published:** 2018-12-08

**Authors:** Tobias Sundberg, Matthew J. Leach, Oliver P. Thomson, Philip Austin, Gary Fryer, Jon Adams

**Affiliations:** 1 Australian Research Centre in Complementary and Integrative Medicine (ARCCIM), Faculty of Health, University of Technology Sydney, Sydney, NSW Australia; 2 Musculoskeletal and Sports Injury Epidemiology Center (MUSIC), Institute of Environmental Medicine, Karolinska Institutet, Stockholm, Sweden; 3 Department of Rural Health, University of South Australia, Adelaide, Australia; 4 University College of Osteopathy, London, UK; 5 Clinical-based Human Research Department, Centre for Osteopathic Medicine Collaboration (COME), Pescara, Italy; 6 Department of Pain Management, Greenwich Hospital, Sydney, Australia; 7 Institute for Health and Sport, Victoria University, Melbourne, Victoria Australia

**Keywords:** Evidence-based practice, Osteopathy, Cross-sectional survey

## Abstract

**Background:**

Evidence-based practice (EBP) is a clinical decision-making framework that supports quality improvement in healthcare. While osteopaths are key providers of musculoskeletal healthcare, the extent to which osteopaths engage in EBP is unclear. Thus, the aim of this cross-sectional study was to investigate UK osteopaths’ attitudes, skills and use of EBP, and perceived barriers and facilitators of EBP uptake.

**Methods:**

UK-registered osteopaths were invited to complete the Evidence-Based Practice Attitude and Utilisation Survey (EBASE) online.

**Results:**

Of the 5200 registered osteopaths in the UK, 9.9% (517/5200) responded to the invitation, and 7.2% (375/5200) completed the EBASE (< 20% incomplete answers). The demographic characteristics of the survey sample were largely similar to those of the UK osteopathy workforce. The osteopaths reported overall positive attitudes towards EBP, with most agreeing that EBP improves the quality of patient care (69.3%) and is necessary for osteopathy practice (76.5%). The majority reported moderate-level skills in EBP, and most (80.8%) were interested in improving these skills. Participating osteopaths typically engaged in EBP activities 1–5 times over the last month. Barriers to EBP uptake included a lack of time and clinical evidence in osteopathy. Main facilitators of EBP included having access to online databases, internet at work, full-text articles, and EBP education materials.

**Conclusions:**

UK osteopaths were generally supportive of evidence-based practice, had moderate-level skills in EBP and engaged in EBP activities infrequently. The development of effective interventions that improve osteopaths’ skills and the incorporation of EBP into clinical practice should be the focus of future research.

## Background

Recent decades have witnessed a gradual movement towards clinical care informed by research evidence [1]. This concept of evidence-based medicine - more inclusively termed evidence-based practice (EBP) - is described as the integration of best available research evidence with clinical expertise and patient values [2], and is now considered a common-sense approach to modern healthcare provision [3]. Despite widespread support for EBP, its integration into healthcare policy and practice has been ad hoc across professions and jurisdictions [4–11]. This may be partly due to a criticism of EBP in housing reliance on algorithm-driven decision-making; challenges in translating research evidence into patient-centred care and difficulties applying EBP to complex clinical presentations may present as additional barriers to EBP uptake [12, 13]. These concerns have been highlighted by various disciplines, including healthcare philosophy [14, 15], medicine [12, 16–19], physiotherapy [20–22], and more recently, osteopathy [23–25].

Osteopathy is a health profession originating in the United States in the late 1800s, where its providers, osteopathic physicians, are licenced to practice in all areas of medicine; yet, although the majority (56%) practice in primary care specialities [26]**,** few (1–2%) specialize in osteopathic manipulative treatment [27]. This is in contrast to osteopathic practitioners, or osteopaths, trained outside the US, where manual therapy treatment may be considered the main scope of osteopathic practice [28]. In the UK, osteopaths are autonomous primary care practitioners primarily trained to manage musculoskeletal conditions, of which spinal pain is the most common, and whom typically provide manual therapy treatment, exercise and self-management recommendations [29, 30].

Osteopathic practice and clinical decision-making is embedded within traditional concepts and principles [31, 32], many of which are drawn from interpretations and observations by prominent individuals made early in the history of the profession. Contemporary research has led some authors to question the validity and usefulness of such models [33–35]. Certainly, there is insufficient research evidence to support all aspects of osteopathy practice and the need for a broader research agenda has been proposed [36, 37]. Although the role of research evidence in osteopathy has been debated, there is agreement that EBP needs to be integrated into the osteopathic approach [23, 38–41]. Nonetheless, potential barriers to the acceptance of EBP by UK osteopaths remain with some for example concerned that implementing EBP may fail to preserve traditional osteopathic principles [13, 42]. Research in physiotherapy points to other factors that may act as barriers to implementing EBP, such as financial and time constraints, and the possible conflict of evidence with patients’ treatment preferences and expectations [43, 44]. Whether these barriers also apply to osteopathy is largely unknown given the relative paucity of EBP research in osteopathy. The aim of the study presented in this paper was to investigate UK osteopaths’ attitudes, skills and utilisation of research evidence in practice, their training in EBP, as well as the barriers to, and facilitators of EBP adoption.

## Methods

### Design and ethics

National cross-sectional survey in the UK. The Research Ethics Committee of the University College of Osteopathy (London, UK) approved the study. No identifying data were collected and results were only reported as aggregate data, thus maintaining participant anonymity. Study participation was voluntary with the option to withdraw/drop-out at any time.

### Setting and participants

All osteopaths registered with the General Osteopathic Council in the UK by June 2017 were eligible and sampled.

### Description of questionnaire and variables

The Evidence-Based practice Attitude and utilization SurvEy (EBASE) is an 84-item instrument evaluating the attitudes, perceived skills and use of EBP amongst healthcare providers [47]. The questionnaire has been previously administered to different health provider groups [4, 5, 8, 9, 47, 48], and psychometric evaluation shows good internal consistency, content validity, construct validity, and acceptable test-retest reliability [47, 49].

EBASE is divided into seven parts: attitude (Part A), skill (Part B), education and training (Part C), use (Part D), barriers to EBP (Part E), and enablers of EBP (Part F). The final section, Part G, gathers information on participant demographics. Parts A, B and D of EBASE generate three subscores: attitude subscore, range from 8 (predominantly strongly disagree) to 40 (predominantly strongly agree); skill subscore, range from 13 (primarily low-level skill) to 65 (primarily high-level skill); and use subscore, range from 0 (mainly infrequent use) to 24 (mainly frequent use). For this study, several survey items were modified for the target population (e.g., the term ‘osteopathy’ was substituted for complementary medicine ‘CAM’). Several response options in Part G (demographics) also underwent change to ensure suitability for a UK audience. These changes did not alter item meaning, and thus, did not affect the validity or reliability of the instrument. The survey was administered electronically (hosted by SurveyMonkey™), with all questions made compulsory to minimise missing data.

### Recruitment and data collection procedures

A pilot study was performed on a convenience sample of five osteopaths associated with the University College of Osteopathy (London, UK), each with varying clinical and academic experience. The purpose of the pilot was to ensure the survey items were clear and appropriate to osteopaths. Some minor terminological changes were made as a result, primarily to enhance clarity. Estimated completion time was 10–15 min.

For the subsequent full study, potential participants were invited to voluntarily participate in the survey through emails sent by the General Osteopathic Council (GOsC), the Institute of Osteopathy (iO) and the National Council for Osteopathic Research (NCOR) in the UK. These agencies also promoted the survey via electronic/paper media. The emails and posts contained a link to EBASE and a participant information sheet, which explained the study, its relevance to the osteopathic profession and what participation would involve. Interested participants had to provide informed consent by responding to a screening question at the beginning of the questionnaire hosted by SurveyMonkey™. A follow-up email was sent two weeks after the initial invitations to remind participants to participate in the study. Data collection was undertaken between the months of June and August 2017. The online survey tool automatically restricted attempts to respond to the survey more than once by the use of cookies per device.

### Statistical methods

The sample size calculation, based on a target population of 5200 osteopaths (March 31, 2017) [45], and a response distribution of 50%, indicated that at least 358 osteopaths would need to complete the EBASE questionnaire to attain a 5% margin of error with a confidence level of 95% for each survey item [46]. Survey responses were exported from SurveyMonkey™ into SPSS (v.24.0) for coding and statistical analysis. Partially-completed surveys, due to drop-out, were excluded from the analysis if more than 20% of all items were incomplete [10]. Any missing data were reported as missing values. Categorical data were described using frequency distributions and percentages. Measures of central tendency and variability were used for normally distributed descriptive data (including continuous [i.e. EBASE subscore] and categorical [i.e. Likert scale] data), while medians and the interquartile range were used to describe non-normally distributed data. Associations between ordinal-level variables were examined using Kendall’s Tau correlation coefficient (Ƭ), and relationships between nominal-level variables assessed using Cramer’s V, with coefficients between 0.10–0.29 representing a weak association, 0.30–0.49 a moderate association, and 0.50 and above a strong correlation. The tests of association were informed by previous research using EBASE [4–6, 8–10]. The level of significance was set at *p* < 0.05.

## Results

A total of 517 (9.9%), out of 5200 UK-registered osteopaths [45] responded to invitations to participate. Excluding 142 responses that were > 20% incomplete, the final response rate was 7.2% (375/5200), which exceeded the minimum required sample size.

### Description of the sample

Participants were largely ≥40 years of age (53.9%), and held an honours degree or higher (54.2%) (Table 1). Participant gender was equal, most had practiced osteopathy for at least 6 years (65.8%), and half worked in southern UK (50.4%). For further demographic data, see Table 1.

**Table 1:** Demographic characteristics of sample (*n* = 375)

Variable	Subcategory	Result
Age, *n (%)*	< 20 years	1 (0.3)
	20–29 years	19 (5.1)
	30–39 years	55 (14.7)
	40–49 years	85 (22.7)
	50–59 years	92 (24.5)
	60–69 years	21 (5.6)
	70+ years	4 (1.1)
	Missing	98 (26.1)
Sex, *n (%)*	Female	146 (38.9)
	Male	130 (34.7)
	Missing	98 (26.1)
Highest qualification, *n (%)*	Certificate	1 (0.3)
	Diploma/Advanced Diploma	34 (9.1)
	Bachelor degree	39 (10.4)
	Honours degree	76 (20.3)
	Graduate Certificate/Diploma	29 (7.7)
	Master’s degree	91 (24.3)
	PhD/Professional doctorate	7 (1.9)
	Missing	98 (26.1)
Years since receiving highest qualification, *n (%)*	< 1 year	15 (4.0)
	1–5 years	42 (11.2)
	6–10 years	79 (21.1)
	11–15 years	41 (10.9)
	16+ years	100 (26.7)
	Missing	98 (26.1)
Years practiced in the field of osteopathy, *n (%)*	< 1 year	7 (1.9)
	1–5 years	23 (6.1)
	6–10 years	68 (18.1)
	11–15 years	56 (14.9)
	16+ years	123 (32.8)
	Missing	98 (26.1)
Hours per week in clinical (osteopathic) practice, *n (%)*	0 h	1 (0.3)
	1–15 h	40 (10.7)
	16–30 h	129 (34.4)
	31–45 h	90 (24.0)
	46+ hours	17 (4.5)
	Missing	98 (26.1)
Hours per week participating in research, *n (%)*	0 h	136 (36.3)
	1–15 h	132 (35.2)
	16–30 h	7 (1.9)
	31–45 h	2 (0.5)
	46+ hours	0 (0.0)
	Missing	98 (26.1)
Hours per week teaching in the higher education sector, *n (%)*	0 h	208 (55.5)
	1–15 h	60 (16.0)
	16–30 h	9 (2.4)
	31–45 h	0 (0.0)
	46+ hours	0 (0.0)
	Missing	98 (26.1)
Treatments typically provided in first osteopathic consultation, *n (%)*	Articulation	255 (68.0)
	Soft tissue therapy	234 (62.4)
	Exercise	226 (60.3)
	HVLA thrust	182 (48.5)
	Muscle energy therapy	179 (47.7)
	General osteopathic treatment	150 (40.0)
	Functional technique	128 (34.1)
	Myofascial release	122 (32.5)
	Relaxation advice	112 (29.9)
	Cranial technique	108 (28.8)
	Ice/cold treatment	83 (22.1)
	Strain-counterstrain	65 (17.3)
	Acupuncture/acupressure	62 (16.5)
	Other	49 (13.1)
	Visceral therapy	43 (11.5)
	Electrotherapy	24 (6.4)
	Orthotics	15 (4.0)
	Steroid injection	1 (0.3)
Clinical setting in which osteopathy is predominantly practiced, *n (%)*	With a group of CAM providers	113 (30.1)
	Solo practice	95 (25.3)
	With CAM & conventional providers	31 (8.3)
	With a group of conventional providers	24 (6.4)
	Within an educational institution	5 (1.3)
	Within a clinical institution	3 (0.8)
	Missing	104 (27.7)
Geographical location*, n (%)*	London	75 (20.0)
	Southeast UK	69 (18.4)
	Southwest UK	45 (12.0)
	Midlands (UK)	20 (5.3)
	Scotland	16 (4.3)
	East Anglia	15 (4.0)
	Northeast UK	14 (3.7)
	Northwest UK	7 (1.9)
	Wales	4 (1.1)
	Other	4 (1.1)
	Northern Ireland	2 (0.5)
	Missing	104 (27.5)
Osteopathy professional association membership, *n (%)*	Institute of Osteopathy	200 (53.3)
	Not a member of an osteopathy association	45 (12.0)
	Sutherland Cranial College of Osteopathy	39 (10.4)
	Other	26 (6.9)
	Sutherland Society	23 (6.1)
	Foundation for Paediatric Osteopathy	18 (4.8)
	Molinari Institute of Health	8 (2.1)
	Rollin E Becker Institute	8 (2.1)
	Institute of Classical Osteopathy	7 (1.9)
Geographical region, *n (%)*	Outer city suburbs	94 (25.1)
	Rural/remote region	70 (18.7)
	City (Central business district)	59 (15.7)
	Inner city suburbs	46 (12.3)
	Missing	106 (28.3)

*CAM* Complementary and alternative medicine, *HVLA* high-velocity low amplitude, *IQR* Interquartile range

### Attitudes toward EBP

Participants reported generally positive attitudes toward EBP, with a median subscore of 30 (IQR 26,33; range 11–40; with a median score ranging between 24.1 and 31.9 defined as a predominantly neutral to agree response). The majority (82.6%) agreed that professional literature and research findings are useful for practice, EBP assists in clinical decision making (80.8%), and EBP is necessary in the practice of osteopathy (76.5%) (Table 2). Most (80.8%) also reported an interest in learning or improving the skills necessary to incorporate EBP into practice.

**Table 2:** Participant attitudes toward evidence-based practice (*n* = 375)

	*1*	*2*	*3*	*4*	*5*	
	Strongly Disagree	Disagree	Neutral	Agree	Strongly Agree	Median (IQR)
	*n (%)*	*n (%)*	*n (%)*	*n (%)*	*n (%)*	
Professional literature (i.e. journals & textbooks) and research findings are useful in my day-to-day practice	2 (0.5)	28 (7.5)	35 (9.3)	**224 (59.7)**	86 (22.9)	4 (4,4)
EBP assists me in making decisions about patient care	8 (2.1)	28 (7.5)	36 (9.6)	**208 (55.5)**	95 (25.3)	4 (4,5)
I am interested in learning or improving the skills necessary to incorporate EBP into my practice	3 (0.8)	29 (7.7)	40 (10.7)	**199 (53.1)**	104 (27.7)	4 (4,5)
EBP is necessary in the practice of osteopathy	8 (2.1)	36 (9.6)	44 (11.7)	**183 (48.8)**	104 (27.7)	4 (4,5)
EBP improves the quality of my patient’s care	8 (2.1)	47 (12.5)	60 (16.0)	**176 (46.9)**	84 (22.4)	4 (3,4)
There is a lack of evidence from clinical trials to support most of the treatments I use in my practice	4 (1.1)	60 (16.0)	67 (17.9)	**176 (46.9)**	68 (18.1)	4 (3,4)
Prioritizing EBP within osteopathic practice is fundamental to the advancement of the profession	33 (8.8)	59 (15.7)	73 (19.5)	**136 (36.3)**	74 (19.7)	4 (3,4)
EBP takes into account my clinical experience when making clinical decisions	11 (2.9)	108 (28.8)	84 (22.4)	**125 (33.3)**	47 (12.5)	3 (2,4)
EBP takes into account a patient’s preference for treatment	20 (5.3)	139 **(37.1)**	90 (24.0)	85 (22.7)	41 (10.9)	3 (2,4)
The adoption of EBP places an unreasonable demand on my practice	35 (9.3)	**181 (48.3)**	99 (26.4)	49 (13.1)	11 (2.9)	2 (2,3)

*EBP* Evidence-based practice, *IQR* Interquartile range

Figures in bold indicate main response

There was no significant association between attitude subscore and most demographic characteristics. There was a weak association between attitude and gender (with higher attitude scores reported in males; *V* = 0.294, *p* < 0.001) and geographical region (with higher attitude scores reported among osteopaths working in the city and inner city suburbs; *V* = − 0.246, *p* < 0.001). There was also a weak negative correlation between attitude and years since receiving highest qualification (Ƭ = − 0.130, *p* = 0.01) and a weak positive correlation between attitude and hours/week participating in research (Ƭ = 0.242, *p* < 0.001).

### Skills in EBP

Participants reported moderate levels of perceived skill in EBP, with a median subscore of 39 (IQR 32,45; range 13–65; with a median score ranging between 26.1 and 39.0 defined as a predominantly low-moderate to moderate skill level). The highest levels of perceived skill in EBP were reported for items relating to clinical problem identification (Table 3). The lowest levels of perceived skill were reported for the conduct of systematic reviews (74.6%) and clinical research (80.5%).

**Table 3:** Participant self-reported skills in evidence-based practice (*n* = 375)

	*1*	*2*	*3*	*4*	*5*		
	Low	Low-moderate	Moderate	Moderate-high	High	Missing	Median (IQR)
	*n (%)*	*n (%)*	*n (%)*	*n (%)*	*n (%)*	*n (%)*	
Identifying precise clinical questions	10 (2.7)	38 (10.1)	**138 (36.8)**	134 (35.7)	55 (14.7)	0 (0.0)	4 (3,4)
Identifying knowledge gaps in practice	7 (1.9)	20 (5.3)	144 (38.4)	**162 (43.2)**	42 (11.2)	0 (0.0)	4 (3,4)
Locating professional literature	16 (4.3)	58 (15.5)	104 (27.7)	**123 (32.8)**	74 (19.7)	0 (0.0)	4 (3,4)
Online database searching	29 (7.7)	81 (21.6)	95 (25.3)	**106 (28.3)**	64 (17.1)	0 (0.0)	3 (2,4)
Retrieving evidence	26 (6.9)	75 (20.0)	**122 (32.5)**	99 (26.4)	53 (14.1)	0 (0.0)	3 (2,4)
Critical appraisal of evidence	24 (6.4)	71 (18.9)	**126 (33.6)**	118 (31.5)	36 (9.6)	0 (0.0)	3 (2,4)
Synthesis of research evidence	40 (10.7)	90 (24.0)	**125 (33.3)**	90 (24.0)	30 (8.0)	0 (0.0)	3 (2,4)
Applying research evidence to patient cases	21 (5.6)	67 (17.9)	**130 (34.7)**	127 (33.9)	30 (8.0)	0 (0.0)	3 (3,4)
Sharing evidence with colleagues	25 (6.7)	91 (24.3)	**114 (30.4)**	100 (26.7)	45 (12.0)	0 (0.0)	3 (2,4)
Using findings from clinical research	24 (6.4)	69 (18.4)	**131 (34.9)**	119 (31.7)	27 (7.2)	5 (1.3)	3 (2,4)
Using findings from systematic reviews	63 (16.8)	83 (22.1)	**120 (32.0)**	82 (21.9)	22 (5.9)	5 (1.3)	3 (2,4)
Conducting systematic reviews	**182 (48.5)**	98 (26.1)	63 (16.8)	15 (4.0)	12 (3.2)	5 (1.3)	2 (1,2)
Conducting clinical research	**213 (56.8)**	89 (23.7)	43 (11.5)	21 (5.6)	9 (2.4)	0 (0.0)	1 (1,2)

*IQR* Interquartile range

Figures in bold indicate main response

There was a weak positive correlation between skill subscore (categorised by quartiles) and highest qualification (Ƭ = 0.240, *p* < 0.001) and hours per week teaching in the higher education sector (Ƭ = 0.212, *p* < 0.001), and a weak negative correlation between skill subscore and years since receiving highest qualification (Ƭ = − 0.204, *p* < 0.001). A moderate positive correlation between skill subscore and hours per week participating in research (Ƭ = 0.382, *p* < 0.001) was also observed.

### Use of EBP

Participants engaged in EBP activities at a moderately-low level in the month preceding the survey, with a median subscore of 12 (IQR 11,15; range 6–30; with a median score ranging between 6.1 and 12.0 defined as a predominantly moderately-low level of use). Most (> 65%) participants partook in the first five EBP-related activities no more than five times in the preceding month (Table 4). A similar level of activity was also reported for consultation with a colleague/industry expert (77.1%) or use of the lay literature (80.6%) to assist clinical decision-making. The only exception to this was the use of online search engines to pursue practice related literature or research, which was performed by 68% of participants, between 1 and 10 times in the month prior.

**Table 4:** Participant use of evidence-based practice (i.e. number of times each activity was performed over the last month) (*n* = 375)

	*1*	*2*	*3*	*4*	*5*		
	0 times	1–5 times	6–10 times	11–15 times	16+ times	Missing	Median (IQR)
	*n (%)*	*n (%)*	*n (%)*	*n (%)*	*n (%)*	*n (%)*	
I have read/reviewed professional literature (i.e. professional journals & textbooks) related to my practice	31 (8.3)	**214 (57.1)**	56 (14.9)	23 (6.1)	32 (8.5)	19 (5.1)	2 (2,3)
I have read/reviewed clinical research findings related to my practice	90 (24.0)	**188 (50.1)**	38 (10.1)	13 (3.5)	27 (7.2)	19 (5.1)	2 (1,2)
I have used professional literature or research findings to assist my clinical decision-making	57 (15.2)	**198 (52.8)**	51 (13.6)	14 (3.7)	36 (9.6)	19 (5.1)	2 (2,3)
I have used an online database to search for practice related literature or research	140 (37.3)	**142 (37.9)**	35 (9.3)	16 (4.3)	17 (4.5)	25 (6.7)	2 (1,2)
I have used professional literature or research findings to change my clinical practice	91 (24.3)	**213 (56.8)**	28 (7.5)	6 (1.6)	18 (4.8)	19 (5.1)	2 (1,2)
I have used an online search engine to search for practice related literature or research	22 (5.9)	**158 (42.1)**	97 (25.9)	32 (8.5)	41 (10.9)	25 (6.7)	2 (2,3)
I have consulted a colleague or industry expert to assist my clinical decision-making	81 (21.6)	**208 (55.5)**	40 (10.7)	6 (1.6)	15 (4.0)	25 (6.7)	2 (2,2)
I have referred to magazines, layperson/self-help books, or non-government/non-education institution websites to assist my clinical decision-making	91 (24.3)	**211 (56.3)**	32 (8.5)	5 (1.3)	11 (2.9)	25 (6.7)	2 (1,2)

*IQR* Interquartile range

Figures in bold indicate main response

There was a moderate positive correlation between use subscore (categorised by quartiles) and hours per week participating in research (Ƭ = 0.300, *p* < 0.001). There was a weak positive correlation between use subscore and highest qualification (Ƭ = 0.226, p < 0.001), hours per week teaching in the higher education sector (Ƭ = 0.120, *p* = 0.032) and geographical region (i.e. higher use scores amongst osteopaths working in the city and inner city suburbs; *V* = 0.174, *p* = 0.005). A weak negative correlation was observed between use subscore and years since receiving highest qualification (Ƭ = − 0.235, *p* < 0.001) and years practicing osteopathy (Ƭ = − 0.133, *p* = 0.013).

Almost one-third (30.7%) of participants reported that no more than 25% of their clinical practice was informed by clinical trial evidence; 20.5% reported clinical evidence informed 26–50% and 51–75% of practice, and 6.4% indicated clinical evidence informed 76–99% of practice. The information source most frequently used by participants to inform their clinical decision-making was traditional knowledge (median rank 3; IQR 1,5), followed by clinical practice guidelines (median rank 4; IQR 2,7) and personal intuition (median rank 5; IQR 2,7) (Table 5).

**Table 5:** Sources of information used to inform clinical decision-making (ranked by most frequent to least frequently used source)^a^ (*n* = 375)

Information source	Median (IQR)
Traditional knowledge	3 (1,5)
Clinical practice guidelines	4 (2,7)
Personal intuition	5 (2,7)
Consulting fellow practitioners or experts	5 (3,7)
Patient preference	5 (3,7)
Personal preference	5 (3,7)
Published clinical evidence (i.e. clinical trials)	6 (3,8)
Textbooks	6 (4,7)
Trial and error	8 (6,9)
Published experimental/laboratory evidence	10 (7,10)

^a^Sources were ranked from 1 = most frequently used, to 10 = least frequently used

### Training in EBP

Most participants reported some level of training in evidence-based practice/osteopathy (81.3%), evidence application (71.5%), critical thinking/analysis (72.3%), and clinical research (57.1%). Participants mainly received this training as a component of a study program (38.7–46.7%), and to a lesser extent, via a seminar or short course (9.6–25.1%). Over half (52.3%) of respondents had received no training in the conduct of systematic reviews and meta-analyses.

### Barriers to and enablers of EBP uptake

The only factors perceived by most participants as being moderate to major barriers to EBP uptake were a lack of clinical evidence in osteopathy (69.1%), and lack of time (56.6%). Most participants perceived other factors to be minor barriers or no barrier to EBP uptake.

Most participants reported that internet in the workplace (70.5%), ability to download full-text articles (60.1%), access to free online databases (55.9%) and online EBP education materials (55.9%) were ‘very useful’ enablers of EBP implementation. Factors considered ‘moderately to very useful’ included access to critical reviews (80.1%), databases requiring licence fees (73.3%), and critically appraised topics relating to osteopathy (67.5%). Very few participants rated the listed enablers of EBP uptake as not useful.

## Discussion

### Key results

#### Response rate and sample characteristics

The response rate for the study was 7.2%. While low, it was within the range (4–8%) of previous national surveys examining EBP use among complementary therapists [5, 8, 10]. However, the response rate was considerably lower than the 30% reported in the UK nationwide Osteopaths’ Opinions Survey in 2012 [50], possibly due to the relatively longer time required to complete the EBASE. Nonetheless, the demographic characteristics of our sample were similar to those of the UK osteopathy profession in terms of gender, age and geographical distribution, years of experience, typical practice setting and types of treatment provided [50, 51]. This suggests that our survey sample was broadly representative of osteopaths in the UK.

#### EBP attitudes

Positive attitudes of UK osteopaths towards EBP support results of previous surveys of CAM professions, including US/Canadian chiropractors [4, 5, 8], western medical herbalists [9], yoga therapists [10], Australian naturopaths and TCM/acupuncture practitioners [6]. Similarly, positive attitudes toward EBP have been reported among physiotherapists [52] and occupational therapists [53].

Thirty-seven per cent of the respondents disagreed that patients’ treatment preferences should be taken into account in EBP, compared with 21–24% of chiropractors [5, 8], and 22% of yoga therapists [10] in North America. This finding suggests UK osteopaths have a limited understanding of EBP and patient-centred care, particularly the awareness of the role of patient’s perspectives among the three main elements of EBP decision-making, alongside best available evidence and the clinician’s expertise [1, 54]. Notwithstanding, most participants agreed or strongly agreed that EBP incorporates clinical expertise into account in clinical decision-making; a view shared by other health professions, especially chiropractors [4, 5, 55]. The implications of these findings to clinical practice require further investigation.

#### EBP skills

Overall, participants reported moderate levels of perceived skill in EBP, with the highest levels reported for items relating to problem identification and evidence acquisition. This suggests that osteopaths perceive themselves as sufficiently skilled in the first two stages of the EBP process, i.e. asking a searchable question and acquiring the right evidence [56]. The findings also indicate that UK osteopaths are moderately skilful in critically appraising, synthesising and applying research evidence to clinical practice, i.e. the final three stages of the EBP process [56]. An implication of these findings may be that clinician training in EBP should focus more on developing skills related to the appraisal and application stages of the EBP process. Indeed, an Australian longitudinal study showed research training for student nurses improved perceived skill level in the latter stages of the EBP process [55].

Participants reported lower levels of skill in using findings from systematic reviews compared to findings from other types of clinical research, which is consistent with previous surveys among chiropractors [4]. The use and interpretability of systematic reviews may be challenging in health professions [57], and given that such studies and meta-analyses represent the highest level of evidence, it is essential that osteopaths gain sufficient skills to utilise such results.

#### EBP use, barriers and facilitators

Although most participants engaged in various EBP-related activities one month preceding the survey, albeit infrequently, almost one-quarter of respondents reported never engaging in EBP activities in the preceding month. This moderately-low level of engagement in EBP-related activities are attributed to several factors, where for example participants cite a lack of time. Participants further reported a perceived lack of clinical evidence in osteopathy as a barrier to EBP uptake; a possible demotivator for practicing osteopaths [58, 59]. Participants indicated that access to the internet at work, online medical databases, full-text journal articles, and online education materials would be very helpful in facilitating EBP uptake. Thus it is likely that inadequate access to these services may significantly affect osteopaths’ ability to engage in EBP [13].

Further, the nature of osteopathic practice in the UK may not impose high-level engagement in EBP-related activities. Perhaps patients seeking care from UK osteopaths present with such a consistent range of symptoms and disorders that osteopaths do not feel the need to engage in EBP activities at a high-level? Such hypothesis may also help explain similar levels of EBP activity within the chiropractic profession [4, 5]. However, the level of engagement in EBP activities of US physical therapists has been reported as higher than that of our cohort, with one study showing 66% of respondents consulting research material and 52% having used a medical database, four to 10 times weekly to make clinical practice decisions [60]. Thus, future research should determine ideal levels of EBP activity for practicing osteopaths, and whether that might vary between different clinical settings and scopes of practice (e.g. Europe vs. US), and further, whether different levels of EBP activity translate into poorer or improved patient outcomes.

#### EBP education

While osteopathic degree courses in the UK provide at least 1000 h of clinical training [51], the extent to which EBP training is embedded within these courses is less clear. The majority (58–81%) of responding UK osteopaths reported some level of training in EBP such as critical thinking/analysis. Most UK osteopaths reported they had undertaken EBP training as a component of a study program, rather than through seminars or short courses.

Almost one-third of participants had been in clinical practice over 16 years. Recognising that Sacket’s EBP model [1] started gaining recognition among healthcare professionals in early 2000, it is probable that some respondents may have received little to no EBP training. Indeed, our analyses showed that the more years in practice, the lower the osteopaths’ EBP attitude and use scores. Similarly, lower EBP use and skill scores correlated with a greater number of years since receiving their highest qualification. Given the current push for EBP in healthcare, and that osteopathic education institutions are facing increasing scrutiny to prepare students to practise osteopathy in a safe and effective manner, effective EBP training and continuing education programs are needed. Notably, our results, which concur with survey findings among yoga therapists [10], suggest that providing opportunities and incentives for clinicians to engage in teaching and research may help to improve EBP uptake.

### Limitations

The low response rate (7.2%) was surprising given that the pilot testing of EBASE did not identify the survey to be too long or time-consuming. However, post-hoc exploratory analysis of the response rates point to possible response fatigue, as several respondents dropped out before completing the survey. However, difficulties completing more complex questions, such as ranking multiple items, is another possible limitation suggested by most drop-outs occurring from question 38 onwards. Other possible explanations for our response rates may be that clinicians were too time-poor to complete the survey (noting that lack of time was a moderate to major barrier to EBP uptake), were generally uninterested in EBP (noting that most osteopaths engaged in EBP activities at a moderately low level), or were not incentivised to participate. Additional study limitations intrinsic to the survey design include recall bias and self-selection bias. Nonetheless, this was the first national survey to comprehensively explore EBP among osteopaths in the UK; the findings of which may be used to inform future research and EBP educational activities for this target group of healthcare providers. The online survey tool automatically restricted attempts to respond to the survey more than once by the use of cookies per device. While it may have been possible to answer the survey multiple times using different devices, the screening of duplicate ISP entries did not reveal any matching demographic data to suggest that any participants completed the survey more than once [61].

## Conclusions

Our study contributes towards an increased understanding of UK osteopaths’ attitudes, skills and use of EBP. The findings suggest that responding UK osteopaths have a generally positive attitude toward EBP, self-report moderate-level skills in EBP, and typically engage in EBP-related activities at a moderately-low level. Encouragingly, most respondents wanted to improve their skills to facilitate the uptake of EBP into osteopathic practice. Additionally, the findings highlight the need for further research; in particular, the need to (i) investigate the meaning that osteopaths ascribe to EBP, (ii) establish the skill level of osteopaths in implementing EBP in clinical practice, (iii) determine a reasonable and clinically feasible level of EBP-related activity for osteopaths, and (iv) develop suitable interventions and strategies that support osteopaths to effectively improve the uptake of EBP.

## References

[1] Sackett DL (1996). Evidence based medicine: what it is and what it isn't. BMJ.

[2] Sackett D (2000). How to practice and teach EBM.

[3] Porta M (2004). Is there life after evidence-based medicine?. J Eval Clin Pract.

[4] Alcantara J, Leach MJ (2015). Chiropractic attitudes and utilization of evidence-based practice: the use of the EBASE questionnaire. EXPLORE: J Sci Heal.

[5] Bussières AE (2015). Self-reported attitudes, skills and use of evidence-based practice among Canadian doctors of chiropractic: a national survey. J Can Chiropr Assoc.

[6] Leach MJ, Gillham D (2011). Are complementary medicine practitioners implementing evidence based practice?. Complement Ther Med.

[7] Mota da Silva T (2015). What do physical therapists think about evidence-based practice? A systematic review. Man Ther.

[8] Schneider MJ (2015). US chiropractors' attitudes, skills and use of evidence-based practice: a cross-sectional national survey. Chiroprc Man Ther.

[9] Snow JE, Leach MJ, Clare BA. Attitudes, skill and use of evidence-based practice among US Western herbal medicine providers: a national survey. J Complement Integr Med. 2017;14(1). 10.1515/jcim-2015-0101 28207415

[10] Sullivan M (2017). Understanding north American yoga therapists' attitudes, skills and use of evidence-based practice: a cross-national survey. Complement Ther Med.

[11] Upton D, Upton P (2006). Knowledge and use of evidence-based practice of GPs and hospital doctors. J Eval Clin Pract.

[12] Greenhalgh T, Howick J, Maskrey N. Evidence based medicine: a movement in crisis? BMJ. 2014;348. 10.1136/bmj.g3725 PMC4056639 24927763

[13] Veziari Y, Leach MJ, Kumar S (2017). Barriers to the conduct and application of research in complementary and alternative medicine: a systematic review. BMC Complement Altern Med.

[14] Loughlin M (2008). Reason, reality and objectivity–shared dogmas and distortions in the way both ‘scientistic’and ‘postmodern’commentators frame the EBM debate. J Eval Clin Pract.

[15] Miles A, Loughlin M, Polychronis A (2008). Evidence-based healthcare, clinical knowledge and the rise of personalised medicine. J Eval Clin Pract.

[16] Hancock HC, Easen PR (2004). Evidence based practice–an incomplete model of the relationship between theory and professional work. J Eval Clin Pract.

[17] Mykhalovskiy E, Weir L (2004). The problem of evidence-based medicine: directions for social science. Soc Sci Med.

[18] Rosenfeld JA (2004). The view of evidence-based medicine from the trenches: liberating or authoritarian?. J Eval Clin Pract.

[19] Tonelli MR (2006). Integrating evidence into clinical practice: an alternative to evidence-based approaches. J Eval Clin Pract.

[20] Bithell C (2000). Evidence-based physiotherapy: some thoughts on ‘best evidence. Physiotherapy.

[21] Herbert RD (2001). Evidence-based practice--imperfect but necessary. Physiother Theory Pract.

[22] Shaw, J.A., D.M. Connelly, and A.A. Zecevic, Pragmatism in practice: mixed methods research for physiotherapy*.* Physiother Theory Pract, 2010(0): p. 1–9. 10.3109/09593981003660222 20649500

[23] Fryer G (2008). Teaching critical thinking in osteopathy–integrating craft knowledge and evidence-informed approaches. Int J Osteopath Med.

[24] Thomson OP, Petty NJ, Moore AP (2011). Clinical reasoning in osteopathy–more than just principles?. Int J Osteopath Med.

[25] Fawkes C, Ward E, Carnes D (2015). What evidence is good evidence? A masterclass in critical appraisal. Int J Osteopath Med.

[26] American Osteopathic Association, 2017 Osteopathic medical profession report, 2017.

[27] American Osteopathic Association. Osteopathic Medical Profession Reports, 2007-2014.

[28] Osteopathic International, A., Osteopathy and Osteopathic Medicine A Global View of Practice, Patients, Education and the Contribution to Healthcare Delivery, 2013.

[29] Fawkes C (2013). Profiling osteopathic practice in the UK using standardised data collection. Int J Osteopath Med.

[30] Fawkes CA (2014). A profile of osteopathic care in private practices in the United Kingdom: a national pilot using standardised data collection. Man Ther.

[31] Cotton A (2013). Osteopathic principles in the modern world. Int J Osteopath Med.

[32] Paulus, S., The core principles of osteopathic philosophy*.* Int J Osteopath Med, 2013. 16(1): p. 11–16.

[33] Hartman SE (2006). Cranial osteopathy: its fate seems clear. Chiropr Osteopathy.

[34] McGrath MC (2015). A global view of osteopathic practice–mirror or echo chamber?. Int J Osteopath Med.

[35] Vogel S (2015). Evidence, theory and variability in osteopathic practice. Int J Osteopath Med.

[36] Steel A (2017). The role of osteopathy in clinical care: broadening the evidence-base. Int J Osteopath Med.

[37] Steel A (2017). Osteopathic manipulative treatment: a systematic review and critical appraisal of comparative effectiveness and health economics research. Musculoskelet Sci Pract.

[38] Vogel S (1994). Research - the future? Why bother?. The British Osteopathic Journal.

[39] Green, J., Evidence-based medicine *or evidence-informed osteopathy?* Osteopathy Today, 2000. April: p. 21–22.

[40] Leach J (2008). Towards an osteopathic understanding of evidence. Int J Osteopath Med.

[41] Licciardone JC (2008). Educating osteopaths to be researchers - what role should research methods and statistics have in an undergraduate curriculum?. Int J Osteopath Med.

[42] Humpage C (2011). Opinions on research and evidence based medicine within the UK osteopathic profession: a thematic analysis of public documents 2003–2009. Int J Osteopath Med.

[43] Poitras S (2012). Guidelines on low back pain disability: interprofessional comparison of use between general practitioners, occupational therapists, and physiotherapists. Spine.

[44] Parr S, May S (2014). Do musculoskeletal physiotherapists believe the NICE guidelines for the management of non-specific LBP are practical and relevant to their practice? A cross sectional survey. Physiotherapy.

[45] General Osteopathic Council, *General Osteopathic Council* Annu Rep *and Accounts 2016–2017*, 2017.

[46] Naing L, Winn T, Rusli B (2006). Practical issues in calculating the sample size for prevalence studies. Medical Statistics.

[47] Leach MJ, Gillham D (2008). Evaluation of the evidence-based practice attitude and utilization SurvEy for complementary and alternative medicine practitioners. J Eval Clin Pract.

[48] Leach MJ (2016). Does ‘traditional’ evidence have a place in contemporary complementary and alternative medicine practice? A case against the value of such evidence. Focus Altern Complement Ther.

[49] Terhorst L (2016). Evaluating the psychometric properties of the evidence-based practice attitude and utilization survey. J Altern Complement Med.

[50] General Osteopathic Council, *GOsC Osteopaths’ Opinion Survey* 2012*:* FINDINGS, 2012.

[51] General Osteopathic Council, Training *courses - General Osteopathic Council.* 2018.

[52] Scurlock-Evans L, Upton P, Upton D (2014). Evidence-based practice in physiotherapy: a systematic review of barriers, enablers and interventions. Physiotherapy.

[53] Dominic U (2014). Occupational Therapists' attitudes, knowledge, and implementation of evidence-based practice: a systematic review of published research. Br J Occup Ther.

[54] Leach MJ (2006). Evidence-based practice: a framework for clinical practice and research design. Int J Nurs Pract.

[55] Leach MJ, Hofmeyer A, Bobridge A (2015). The impact of research education on student nurse attitude, skill and uptake of evidence-based practice: a descriptive longitudinal survey - leach - 2015 - journal of clinical nursing - Wiley online library. J Clin Nurs.

[56] de Groot M (2013). Evidence-based practice for individuals or groups: let’s make a difference. Perspect Med Educ.

[57] Murthy, L., et al., *Interventions to improve the use of systematic reviews in decision-making by health system managers, policy makers and clinicians*, 2012. 10.1002/14651858.CD009401.pub2 PMC11627148 22972142

[58] Leach MJ, Tucker B (2017). Current understandings of the research–practice gap from the viewpoint of complementary medicine academics: a mixed-method investigation. EXPLORE: J Sci Heal.

[59] Wardle J, Adams J (2013). Are the CAM professions engaging in high-level health and medical research? Trends in publicly funded complementary medicine research grants in Australia. Complement Ther Med.

[60] Fell DW, Burnham JF, Dockery JM (2013). Determining where physical therapists get information to support clinical practice decisions. Health Info Librar J.

[61] Konstan JA, Simon Rosser BR, Ross MW, Stanton J, Edwards WM (2005). The story of subject naught: a cautionary but optimistic tale of internet survey research. J Comput-Mediat Commun.

